
JOURNAL INFORMATION
==============================
NLM Title Abbreviation: Wirtschaftsdienst
Journal ID: Wirtschaftsdienst
NLM Journal ID: 101086612

Wirtschaftsdienst (Hamburg, Germany : 1949)

ISSN: 0043-6275

ARTICLE INFORMATION
==============================
PMCID: PMC7896163
PMID: 33642641
DOI: 10.1007/s10273-021-2841-5
Article ID: 2841
Article version: 1
Subjects: Kurz Kommentiert

Daten gegen Steuervermeidung?

Steuerliches Country-by-Country-Reporting

Tax Country-by-Country Reporting: Data Against Tax Avoidance?

Langenmayr Dominika dominika.langenmayr@ku.de

grid.440923.8 0000 0001 1245 5350 Wirtschaftswissenschaftliche Fakultät, Katholische Universität Eichstätt-Ingolstadt, Auf der Schanz 49, 85049 Ingolstadt, Deutschland
Electronic publication date: 2021 Feb 20
Print publication date: 2021
Volume: 101
Issue: 2
First page: 74
Last page: 74
Copyright: © Der/die Autor:in(nen) 2021
License: Open Access: Dieser Artikel wird unter der Creative Commons Namensnennung 4.0 International Lizenz veröffentlicht (http://creativecommons.org/licenses/by/4.0/deed.de).
Open Access wird durch die ZBW–Leibniz-Informationszentrum Wirtschaft gefördert.
License URL: https://creativecommons.org/licenses/by/4.0/

issue-copyright-statement: © The Author(s) 2021

==============================
DOI: 10.1007/s10273-021-2841-5

Steuerliches Country-by-Country-Reporting

### Daten gegen Steuervermeidung?

Wie in jeder Krise gibt es auch in der Covid-19-Pandemie einige Gruppen, denen die krisenbedingten Änderungen nützen. Eine solche Gruppe ist der Online-Versandhandel, auf den viele Konsument:innen aufgrund von Lockdowns und Angst vor Infektionen ausweichen. Es ist somit wenig überraschend, dass die Besteuerung von Unternehmen wie Amazon zunehmend in den Fokus gerät. In diese Richtung zielt auch der Vorstoß der portugiesischen EU-Ratspräsidentschaft, dass multinationale Unternehmen ihre Steuerdaten auf Länderbasis veröffentlichen sollen.

Im Grundsatz ist dies kein neuer Vorschlag. Bereits seit 2016 müssen große multinationale Unternehmen Informationen zu ihren Aktivitäten auf Länderebene zusammenstellen. Die Daten aus diesen Country-by-Country-Reportings gehen bisher nur an die Finanzverwaltung; die Öffentlichkeit erhält nur Zusammenfassungen auf Länderebene. Bereits vor der Einführung des Country-by-Country-Reporting wurde gefordert, die Daten einzelner Unternehmen zu veröffentlichen. Die portugiesische EU-Ratspräsidentschaft bringt diese Forderung nun erneut auf. Größere Transparenz sei ein wichtiger Schritt zur Bekämpfung der Steuervermeidungstätigkeiten multinationaler Unternehmen.

Um was für Daten geht es? Die Country-by-Country-Reportings enthalten vor allem Informationen zu Umsatz, Gewinn vor Steuern, gezahlten Gewinnsteuern, Anlagevermögen und Beschäftigtenzahl in jedem Land, in dem das Unternehmen aktiv ist. Laut geltendem Recht müssen Konzerne mit einem Jahresumsatz über 750 Mio. Euro einen solchen Bericht für die Steuerbehörde ihres Sitzlandes erstellen. Von dort werden die Informationen an die Steuerbehörden in allen anderen Ländern weitergegeben, in denen der Konzern aktiv ist und die sich am Country-by-Country-Reporting beteiligen – momentan 90 Länder weltweit; alle EU-Staaten sind dabei.

Bisher dürfen diese Daten also nur von der Finanzverwaltung genutzt werden. Dies liegt vor allem daran, dass Wirtschaftsvertreter:innen seit Jahren gegen eine Veröffentlichung der Daten argumentieren. Sie führen an, dass Konkurrenz (beispielsweise aus China) die Daten nutzen könnte, um die Profitabilität ihrer deutschen Wettbewerber zu sezieren und sie dann mit Kampfpreisen aus dem Markt zu drängen. Dieses Argument überzeugt nicht: Der Jahresabschluss aller deutschen Kapitalgesellschaften und größeren Personengesellschaften muss sowieso im Bundesanzeiger veröffentlicht werden. Trotzdem zollt der neue EU-Vorschlag diesem Einwand Rechnung: Der Vorschlag Portugals sieht eine Ausnahmeregelung für sensible Informationen vor und ermöglicht, diese sechs Jahre lang zurückzuhalten.

Was wäre der Vorteil, wenn die Daten zu einzelnen Firmen veröffentlicht würden? Dies könnte zu öffentlichem Druck auf Unternehmen führen, „faire" Steuern zu zahlen: Das heißt, in den Ländern Steuern zu zahlen, in denen sie aktiv sind, und Gewinne nicht in Steueroasen zu verschieben.

Dies setzt voraus, dass über diese Daten angemessen berichtet wird und die Berichtenden die Feinheiten verstehen, durch die sehr niedrige Steuerzahlungen entstehen können: Beispielsweise ist es möglich, dass ein Unternehmen trotz hoher Umsätze in Deutschland keine Steuern zahlt, da es in der Vergangenheit nicht profitabel war und nun Verlustvorträge nutzen kann. Dies ist keine Steuervermeidung, sondern von der Gesetzgebung so vorgesehen und für eine gerechte Unternehmensbesteuerung sogar notwendig. Aufgrund der Komplexität des Steuerrechts ist es nicht immer einfach, solche Sachverhalte korrekt einzuordnen. Das Risiko, dass über Unternehmen zu Unrecht sehr negativ berichtet wird, ist also nicht abzustreiten.

Eine Lösung wäre, nur ausgewählten Personen – etwa aus der Wissenschaft – unter Auflagen den Zugang zu den Daten zu gewähren. Sie könnten mit Verständnis und Zeit die Daten analysieren. Für die Analysen könnten Einzeldaten genutzt werden, veröffentlicht würden aber nur Studienergebnisse, die keinen Rückschluss auf einzelne Unternehmen zulassen. So würden zwar keine einzelnen Unternehmen an den Pranger gestellt, Studien könnten aber z. B. aufzeigen, in welchen Branchen besonders aggressiv Steuern vermieden werden. Auch könnten so die Wirksamkeit von gesetzlichen Gegenmaßnahmen untersucht und evidenzbasierte Vorschläge zur Bekämpfung von Steuervermeidung erarbeitet werden. Würde man den Wissenschaftler:innen zudem ermöglichen, verschiedenen Datenquellen zu verknüpfen, wäre auch eine zielgenauere Identifikation von Steuervermeidung möglich, als es den Finanzämtern heute mit den wenigen Informationen aus dem Country-by-Country-Reporting alleine möglich ist. Gleichzeitig würden der Datenschutz und das Steuergeheimnis gewahrt werden.

Dominika Langenmayr
Katholische Universität Eichstätt-Ingolstadt
dominika.langenmayr@ku.de

© Der/die Autor:in(nen) 2021. Open Access: Dieser Artikel wird unter der Creative Commons Namensnennung 4.0 International Lizenz veröffentlicht (creativecommons.org/licenses/by/4.0/deed.de).

Open Access wird durch die ZBW – Leibniz-Informationszentrum Wirtschaft gefördert.
